# Host Ubiquitin-Specific Proteases Regulate All Stages of Herpesvirus Infection

**DOI:** 10.3390/ijms27219631

**Published:** 2026-10-28

**Authors:** Mohamed Maarouf, Christopher S. Peterson, Kareem N. Mohni

**Affiliations:** Department of Molecular Medicine, Mayo Clinic, Rochester, MN 55905, USA

**Keywords:** ubiquitin-specific proteases, ubiquitin signaling, herpesvirus replication, DNA damage response, replication stress, genome maintenance, antiviral defense, herpesviruses

## Abstract

Herpesviruses are a large group of double-stranded-DNA viruses that replicate their genomes in the nucleus, forming large replication compartments during lytic infection. Herpesviruses are known to modulate host DNA replication and DNA damage-repair proteins to promote their replication. Ubiquitination is a cellular process that can alter protein stability, localization, and availability, including that of multiple proteins involved in genome maintenance. Ubiquitin-specific proteases (USPs) are a class of deubiquitinating enzymes (DUBs) that counter this process by removing ubiquitin chains, thus acting as another level of regulation. Proteins from each of the three herpesvirus subfamilies (alpha-, beta-, and gammaherpesviruses) have been reported to interact with at least one host USP, and USP7 in particular is reported as a target in representatives of all three subfamilies. In this review, we give an up-to-date summary of herpesviruses–USPs interactions, with special emphasis on DNA replication, and how these interactions could shape infection outcomes. We also examine published proteomic datasets for herpesvirus-driven host-proteome modulation and offer a framework for future directions to prioritize potentially important, understudied USPs. Lastly, we visit herpesvirus-encoded DUBs and highlight their roles in viral replication by remodeling ubiquitin signaling, discussing viral DUBs and virus–USP interfaces as proposed antiviral targets.

## 1. Introduction

Ubiquitination is a reversible post-translational modification that modulates protein stability, localization, and signaling by attaching ubiquitin (Ub) chains to target proteins in diverse chain architectures. Three types of enzymes, E1 ubiquitin-activating enzyme, E2 ubiquitin-conjugating enzyme, and E3 ubiquitin ligase, perform this function, and the sequential cascades of E1–E2–E3 enzymes have been well reported [1,2,3,4]. Ubiquitin is a 76-amino-acid protein highly conserved across eukaryotes [5]. Protein ubiquitination is essential for the regulation of various eukaryotic processes [6]. Ubiquitin contains seven lysine (K) residues (K6, K11, K27, K29, K33, K48, and K63) and an N-terminal methionine (Met1) that can each serve as linkage points for chain formation, resulting in multiple polyubiquitin linkage types. The linkage composition, chain architecture (including mixed or branched chains), and chain length collectively influence the fate and signaling output of the modified target protein [5]. For instance, K11- and K48-linked polyubiquitin chains lead to proteasome-mediated degradation of the protein substrate via the Ub–proteasome system [7,8], while K63-linked chains are mainly involved in non-degradative roles in cellular pathways including intracellular trafficking of linked proteins, DNA repair, and immune responses [9,10,11,12]. It has been estimated that at least 80% of protein degradation in mammalian cells occurs through the Ub–proteasome system [13].

Meanwhile, deubiquitination is the counteracting process catalyzed by deubiquitinating enzymes (DUB), which hydrolyze Ub–substrate and Ub–Ub peptide or isopeptide bonds and can thereby reverse or reshape the ubiquitin-dependent fate of target proteins [4,14]. The centrality of ubiquitin-dependent regulation to cell biology was highlighted by the 2004 Nobel Prize in Chemistry for the discovery of ubiquitin-mediated protein degradation. The human genome encodes approximately 100 DUBs, which can be divided into seven families: one Zn^2+^-dependent metalloprotease family and six cysteine protease families. The metalloprotease family contains the JAB1/MPN/MOV34 metalloenzyme (JAMM/MPN^+^) family, whose catalytic mechanism requires a coordinated metal ion [15,16]. Cysteine protease DUBs are further classified into six major superfamily groups: (1) the Ub-specific protease (USP/ubiquitin-specific processing protease (UBP)) [17], which is the largest superfamily of DUBs; (2) the ovarian tumor proteases (OTUs) [18]; (3) the Machado–Josephin domain proteases (MJD) [19]; (4) the Ub C-terminal hydrolases (UCHs) [20]; (5) MINDY-family DUBs [21], and (6) Zinc Finger With UFM1-Specific Peptidase Domain Protein (ZUFSP/ZUP1) [22,23,24,25,26].

Ubiquitin signaling is a core regulator of various cellular processes, including the DNA damage response (DDR). DUBs, on the other hand, edit ubiquitin modifications to tune protein fate and modify the engagement of repair pathways [27]. Ubiquitin-specific proteases (USPs) are the largest family of human deubiquitinases, with approximately 56 reported USPs [28], and they modulate numerous cellular processes and signaling pathways, including DDR signaling, cellular differentiation, epigenetic regulation, and protein stability [29,30]. For instance, USP1 negatively regulates the Fanconi anemia (FA) DNA repair pathway by deubiquitinating FANCD2, and USP1 associated factor 1 (UAF1) was found to be a stoichiometric USP1 partner in a multi-subunit protein complex that regulates this pathway [31]. Herpesviruses have also been reported to interact with and/or interfere with ubiquitin signaling in ways that could modulate DDR pathways.

Recent reports have identified direct USP–virus interactions at key steps in viral replication and infection, including at viral replication origins, on nascent viral DNA, in DNA-sensing pathways, and in stabilizing viral proteins. In this review we highlight the current evidence for how herpesviruses co-opt defined USP pathways to support or hinder viral replication or fork progression, and to stabilize viral proteins, and how viral DUBs can mimic or counteract host ubiquitin editing to support infection. We conclude that these well-supported herpesvirus–USP examples provide a solid framework for broader investigation. Major gaps remain in pinpointing which USPs are truly rate-limiting across virus families and cell types, and in determining when targeting USP activity could yield antiviral benefit without unacceptable genome instability or immunologic impacts.

## 2. Ubiquitin-Specific Proteases (USPs) in Genome Maintenance

### 2.1. USP Architecture and Regulation

USPs are the largest DUB subfamily and adopt a conserved hand-like architecture with palm–thumb–fingers subdomains. The catalytic center lies at the interface between the palm and the thumb, while the fingers engage ubiquitin and help position its C-terminus into the catalytic cleft for hydrolysis of the Ub C-terminal linkage [4,32]. USPs also include various modules at the N- and/or C-terminus that contribute to their substrate specificity, recruitment, and localization. These modules can include TRAF-like domains, ubiquitin-like (UBL) domains, zinc-binding motifs, and large insertions within the catalytic domain [4,33]. UBL domains adopt a ubiquitin-like fold, where they can support intramolecular regulation, partner binding, or subcellular targeting [34]. UBL domains can exist as single or multiple copies in a USP and serve a variety of functions. These can range from driving the localization of USPs to the proteasome, such as with USP14, to autoregulatory functions, such as in USP4, where the UBL domain binds its own catalytic domain, decreasing activity [34].

Many USPs are regulated by conformational changes and cofactor binding that are required for their full catalytic activity [5]. Allosteric regulation is another key layer of USP activity modulation. For instance, the last two UBL domains of USP7 were reported to be sufficient to activate USP7, with an adjacent C-terminal activation peptide being required for full activation. This activity is further enhanced allosterically by GMPS binding to the first three UBL domains (HUBL-123) of USP7 to induce the hyperactivation of USP7 [35]. USPs can also be activated allosterically by binding cofactors at distinct sites distant from the catalytic center, as in UAF1 (WDR48) and WDR20 β-propeller cofactors activation of USP12 [36]. In short, USP regulation can occur through intramolecular mechanisms, where domains within the USP allosterically tune catalytic activity, and through external mechanisms, where post-translational modifications, dedicated adaptor proteins, substrate-assisted conformational activation, and partner complexes modulate USP activity and substrate affinity [37].

### 2.2. Roles of Key USPs in DNA Damage Signaling and Replication Fork Progression

Ubiquitin signaling is crucial for genome stability, and USPs act as editors by removing or remodeling ubiquitin [27,33]. Multiple USPs have also been reported to play roles at stalled forks and DNA damage lesions. A well-defined genome-maintenance module is the USP1–UAF1 complex in the Fanconi Anemia (FA) pathway and replication-associated repair. RNAi library screening identified USP1 as an FA-pathway component, and USP1 depletion resulted in the hyperaccumulation of monoubiquitinated FANCD2. USP1 was found to physically associate with FANCD2, with chromatin colocalization after DNA damage [38]. A follow-up study showed that UAF1 is a stoichiometric activator of USP1 that increases catalytic turnover (k_cat_) without increasing substrate affinity (K_M_), and DNA damage was found to cause immediate transcriptional shutoff of the USP1 gene, resulting in a rapid decline in the USP1/UAF1 complex [31]. USP1 also deubiquitinates PCNA to restrain translesion synthesis (TLS)-associated damage tolerance pathways [39]. The UV-induced autocleavage of USP1 resulted in increased monoubiquitinated PCNA, which promotes TLS [40].

USP7 was also found to be involved in DNA replication progression. Minichromosome maintenance binding protein (MCM-BP) directly interacts with USP7 on chromatin, tethering USP7 to MCM proteins. The knockout of USP7 slowed late S-phase progression and impaired the dissociation of MCM proteins from chromatin in mid- to late S phase [41]. USP37 also aids in replication by deubiquitinating MCM7, thus stabilizing the replication helicase and preventing its disassembly [42].

In homologous recombination (HR), the heterodimer BRCA1/BARD1 is important for response to DNA double-strand breaks (DSBs), both through its E3 ligase function when ubiquitinating, and thus deactivating, RNA pol II, and through antagonistic competition with 53BP1 to direct repair down the homologous recombination pathway [43,44,45]. USP15 was also found to promote homologous recombination (HR) as MDC1 recruits USP15 to DSBs to deubiquitinate the BRCT domain of BARD1, promoting BARD1–HP1γ interaction and BRCA1/BARD1 retention at DSBs [46]. USP11 was also reported to interact with BRCA2 and to participate in DDR functions within the BRCA2 pathway; notably, a catalytically inactive USP11 retained interaction with BRCA2, and USP11 expression did not affect BRCA2 ubiquitination or protein levels [47]. USP28 dimerization was reported to be regulated by 53BP1, which binds and stabilizes USP28 dimers; genotoxic stress diminishes 53BP1–USP28 interaction, promoting USP28 monomerization and aberrant DNA synthesis associated with replication-associated DNA damage [48].

### 2.3. USPs Shape Cell-Cycle Decisions

USPs are integral regulators of cell cycle progression, as exemplified by the interaction between USP7 and the p53–MDM2 pathway. MDM2 is an E3 Ub ligase that targets p53 for degradation. MDM2 also catalyzes its own ubiquitination, which is counteracted by USP7. The loss of USP7 results in rapid MDM2 degradation and stabilization and the activation of p53 [49]. Structural work mapped the USP7 interaction site for p53 and MDM2 to the N-terminal domain of USP7, and showed that p53 and MDM2 bind competing sites on this domain. Epstein–Barr virus nuclear antigen 1 (EBNA1) also uses the same N-terminal binding pocket, consistent with competitive binding [50]. USP10, a predominantly cytoplasmic USP, translocates to the nucleus following DNA damage to deubiquitinate p53 and counter MDM2-induced nuclear export and degradation [51].

USPs can also be cell cycle-regulated. For example, USP37 expression begins during G1, peaks at the G1/S threshold, and is degraded during mitosis. USP37 binds CDH1 to deubiquitinate cyclin A. Ectopic USP37 expression accelerated S-phase entry after premature cyclin A accumulation in G1 [52]. USP37 also regulates CHK1 stability, and USP37-depleted cells showed higher sensitivity to replication stress and higher levels of DNA damage markers γH2AX and 53BP1 [53].

**Figure 1:**
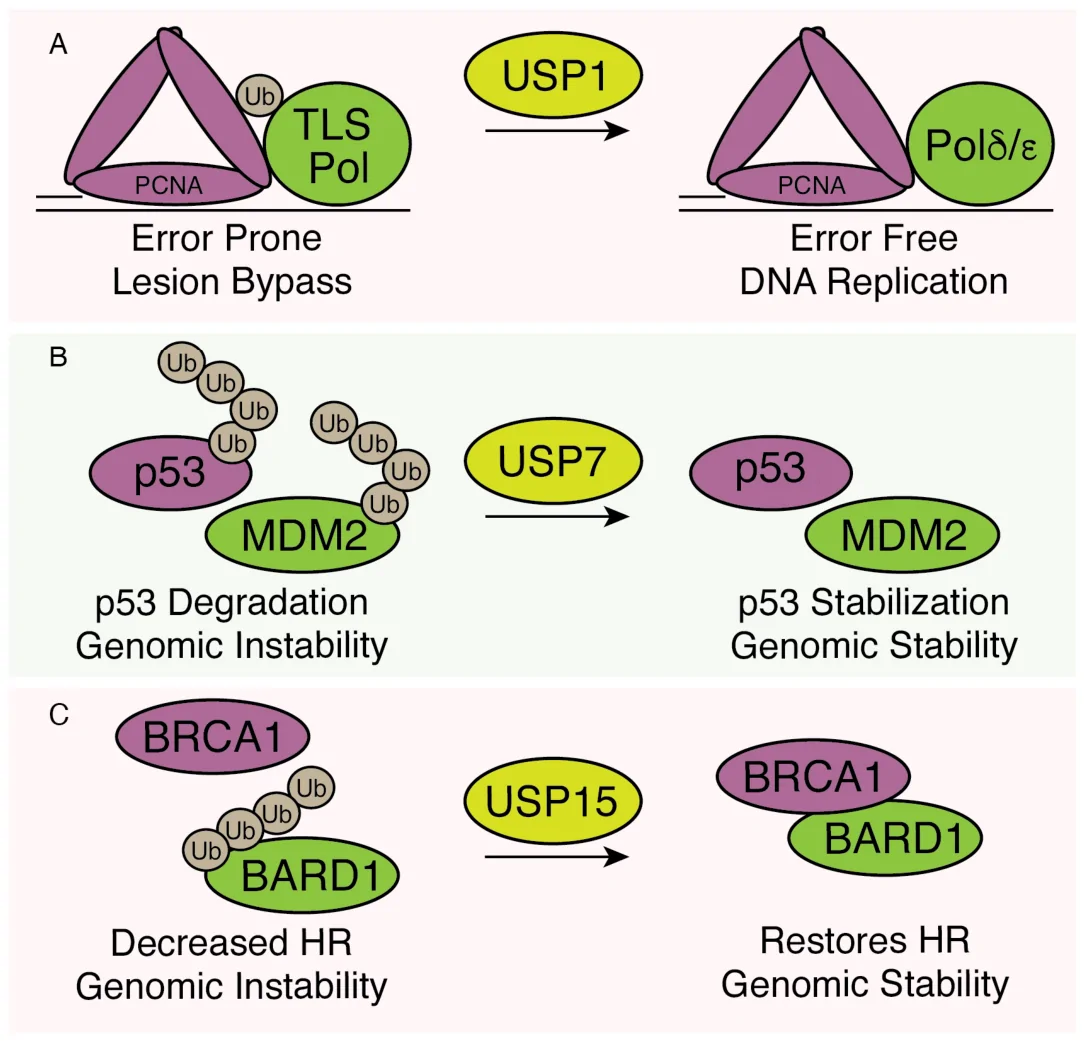
Cellular functions of select USPs. Schematic summary demonstrating the cellular functions of USP1, USP7, and USP15. (**A**) PCNA is ubiquitinated to promote the recruitment of error-prone TLS polymerases to bypass DNA lesions. USP1 removes the ubiquitin on PCNA to restore the interaction with error-free replicative polymerases. (**B**) MDM2 ubiquitinates itself and p53, promoting the rapid turnover of both proteins. USP7 removes the ubiquitin to stabilize p53 to promote genomic stability. (**C**) Ubiquitination of BARD1 prevents the interaction with BRCA1. USP15 functions to remove the ubiquitin and promote the association of BRCA1 and BARD1 to promote homologous recombination (HR) and the error-free repair of DNA breaks.

The cellular functions of several USPs are highlighted in Figure 1.

## 3. Ubiquitin-Specific Proteases (USPs) in Herpesvirus Infection

Herpesviruses are large double-strand DNA viruses that establish the life-long infection of their hosts. As nuclear replicating viruses, they must contend with many areas of cell biology regulated by DUBs such as DNA replication, cell cycle control, and DNA repair. We have selected several representative examples of how herpesvirus infections interact with host DUBs and their relevant impact on viral replication, viral protein stability, and overall infection outcome.

To identify these examples, we searched PubMed and Google Scholar with terms including herpesvirus in combination with proteomics, phosphoproteomics, deubiquitinase, ubiquitin-specific protease, iPOND, and aniPOND. No limit was placed on publication date. We retained studies that reported quantitative measurements of protein abundance, phosphorylation, or association with viral DNA in infected cells, and that made those data available in the paper or its supplementary files. Each retained dataset was then examined for USPs showing altered abundance, altered phosphorylation, or enrichment on viral DNA.

### 3.1. HSV-1: ICP0–USP7 and UL12-USP15

The HSV-1 immediate early protein ICP0 is an E3 ubiquitin ligase that functions by degrading innate antiviral proteins to promote lytic infection [54]. Early studies searching for ICP0-interacting proteins identified a host protein of 135 kDa that bound to the C-terminal region of ICP0 [55]. This protein was later identified as USP7 [56]. The binding was mapped to a short region comprising amino acids 594–633, and an ICP0 mutant with an internal deletion in the region failed to interact with USP7 [57]. Viruses expressing ICP0 mutants that cannot interact with USP7 are unable to stimulate the expression of viral promoters and are attenuated in viral growth [58]. It was later discovered that USP7 was promoting the stabilization of ICP0 by counteracting ICP0-mediated autoubiquitination [59]. Conversely, ICP0 also targets USP7 for degradation [60]. Together, these data indicate that ICP0 and USP7 interaction is responsible for fine-tuning the amount of ICP0 needed for a productive infection and maintaining necessary functions of ICP0 in stimulating gene expression and the correct targeting of cellular proteins.

Subsequent structural work described exactly how ICP0 and USP7 interact. USP7 is a large protein of 1102 amino acids that contains seven domains. These include the N-terminal TRAF-like and catalytic domains as well as five C-terminal UBL domains (UBL1-UBL5) [61]. Biochemical works with protease resistant fragments of USP7 indicate that the C-terminal regions of USP7 are necessary for ICP0 interaction [62]. Two complementary structural papers identified a loop in USP7 UBL2 that binds ICP0. A crystal structure of the UBL1-UBL3 with an ICP0 positively charged peptide encoding amino acids 617–627 showed that ICP0 binds to a negatively charged region in UBL2. This featured salt bridges between ICP0 K620 and K624 with USP7 D762 and D764 [63]. A second study solved solution NMR structures of UBL1, UBL1-UBL2, and UBL3, titrated in ICP0 peptide 617–627, and looked for peak changes in the NMR spectra. They only observed peak changes in UBL1-UBL2 and not in UBL1 or UBL3. They also utilized isothermal calorimetry to identify that UBL1-UBL2 binds ICP0 with a 1:1 stoichiometry [64]. Together, these studies define the exact molecular interactions between ICP0 and USP7 that could be targeted for therapeutic intervention.

Proteomic studies of replicating HSV-1 DNA identified USP15 as one of the most enriched cellular proteins on nascent DNA [65]. HSV-1 encodes an alkaline nuclease, UL12, that together with ICP8 forms a two-subunit recombinase necessary for homology-directed repair [66]. Proteomic analysis of replicating DNA in a ΔUL12 virus found the complete absence of USP15 associated with DNA. Follow-up studies identified that UL12 directly interacts with USP15 and recruits USP15 to viral DNA [67,68]. USP15 is required for UL12 stability as infection of a ΔUSP15 cell line resulted in reduced amounts of UL12 and a significant reduction in virus yield [68]. UL12 also failed to stimulate the repair of a single strand annealing (SSA) reporter assay in the ΔUSP15 cell line. UL12 protein levels could be rescued with WT and catalytic dead USP15, but SSA was only rescued with WT protein. These data indicate that both WT and catalytic dead USP15 stabilize UL12, but only WT USP15 can rescue single strand annealing, indicating that USP15 likely has other unknown targets necessary for single strand annealing [68]. Biochemical studies mapped the interaction of USP15 to a C-terminal alpha helix in UL12. This helix is conserved in another alphaherpesvirus, PRV UL12, but not in the gammaherpesvirus KSHV SOX protein. Mutations in the UL12 helix abrogate the interaction with USP15 and fail to recruit USP15 [69]. Thus, USP15 is required for efficient replication and recombination during HSV-1 infection.

### 3.2. HCMV: UL35–USP7 and UL138–UAF1–USP1

The HCMV UL35 gene is required for viral gene expression and efficient replication. The UL35 gene codes for two proteins, UL35 and UL35a. Mass spectrometry analysis of UL35/UL35a-interacting proteins identified USP7 as an interactor of both proteins [70]. UL35 expression was sufficient to alter USP7 subcellular localization. Another interacting protein complex, Cul4^DCAF1^ E3 ubiquitin ligase complex (DCAF1, DDB1, and DDA1), interacts with UL35, but not UL35a. Mapping with DCAF1 mutants further indicated that the C-terminal region of DCAF1 was required for association with UL35 and mediated the relocalization of DCAF1 into UL35 nuclear bodies [70].

A search for protein interactors of HCMV UL138, a pro-latency protein, identified WDR48 (UAF1) [71]. UAF1 is a scaffold protein necessary for the activity of USP1, USP12, and USP46. UL138, UAF1, and USP1 were all confirmed to interact during HCMV infection. USP1 was required for the continued phosphorylation of STAT1 and for the establishment of latency [71]. USP1-UAF1 are known to regulate the ubiquitination status of PCNA and FANCD2. A follow-up study found that USP1, PCNA, and FANCD2 were all recruited to sites of viral DNA synthesis [72]. The knockdown of USP1 showed a significant increase in virus yields, indicating that USP1 restricts viral replication. Mechanistically, USP1, in conjunction with PCNA and FANCD2, contributes to the genomic integrity of replicating HCMV DNA [72]. Control of the ubiquitination status of PCNA is also important for HCMV genomic integrity as monoubiquitinated PCNA is critical for the recruitment of TLS polymerases, which also promote the integrity of the HCMV genome [73].

### 3.3. EBV and KSHV: EBNA1–USP7 and vIRF1-USP7

The EBV EBNA1 protein is an essential protein for the replication and maintenance of the latent viral genome. A search for EBNA1-interacting proteins identified USP7 and the interaction was mapped to the N-terminal p53 binding domain of USP7 [62,74]. The interaction with USP7 requires amino acids 436–450 of EBNA1 [75]. This EBNA1 peptide was cocrystalized with the USP7 N-terminal domain. EBNA1 bound to a shallow depression in the surface of USP7 and the interaction was mediated by a combination of hydrophobic and hydrophilic interactions. NMR chemical shift analysis indicated that EBNA1 and p53 compete for the same binding site on USP7. Functionally, this acts to reduce the levels of cellular p53 and make cells more resistant to apoptosis [75]. EBNA1 is also able to cause the disruption of PML nuclear bodies. This disruption requires the interaction with USP7. This disruption impairs the induction of p53, DNA repair, and apoptosis following DNA damage [76].

The KSHV vIRF1, encoded by ORF K9, has homology to cellular IRF3 and IRF7 and functions by disrupting cellular antiviral activity. vIRF interacts with USP7 through a peptide motif of EGPS located at the N-terminus of vIRF. This amino acid motif is identical to the EBNA1 motif required to interact with USP7. Indeed, the cocrystal of vIRF1 peptide with the USP7 N-terminal domain revealed an identical mode of binding to that of EBNA1-USP7 [77]. KSHV vIRF1 and ORF45 also disrupt p53 signaling by competing for binding with USP7 and therefore inducing the destabilization of p53 [77,78]. vIRF2, vIRF3, and vIRF4 also interact with USP7 to regulate innate immune sensing and latency [79,80,81].

**Figure 2:**
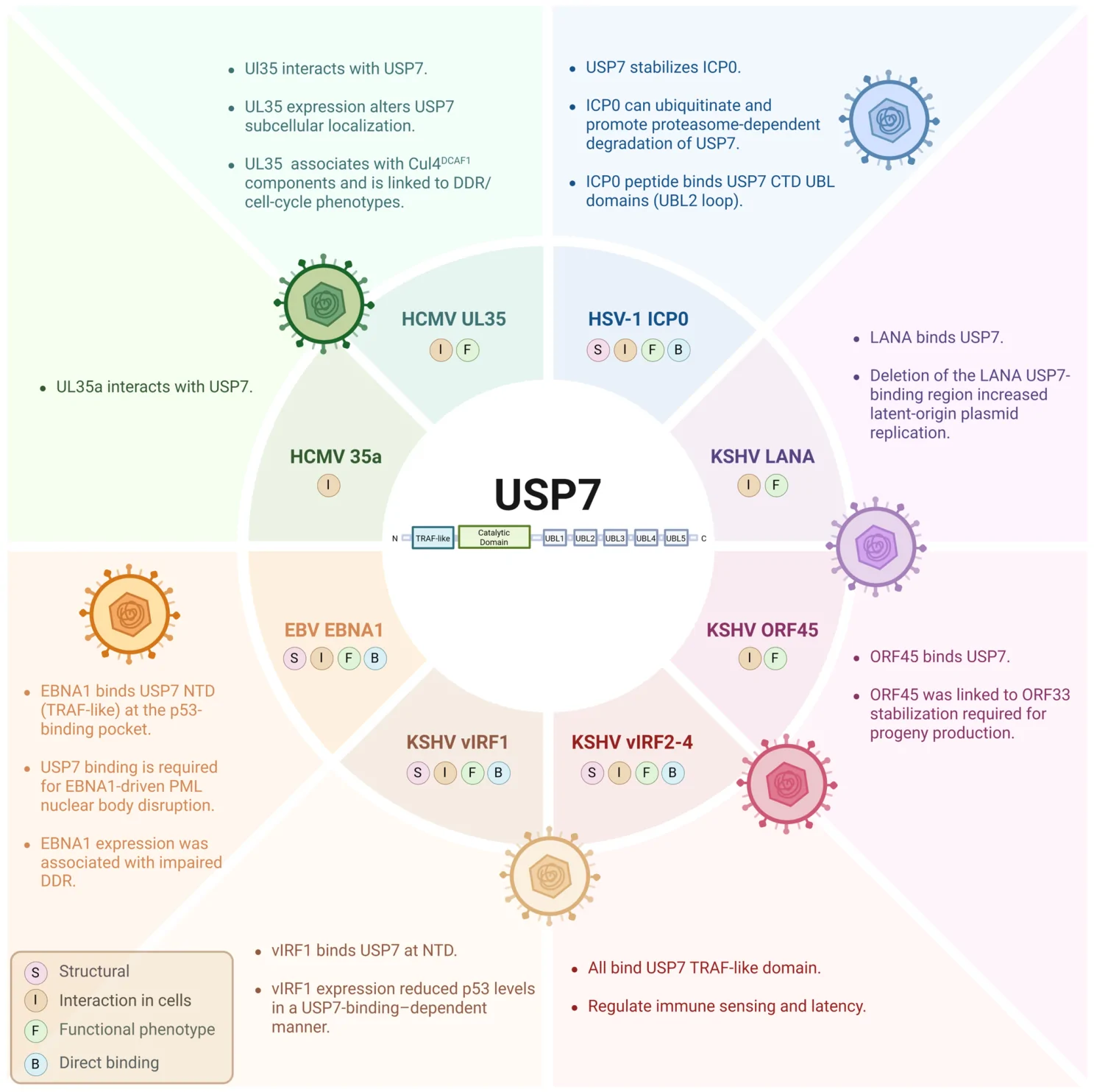
USP7 interfaces targeted by herpesviruses. Schematic summary of reported interactions between host ubiquitin-specific protease 7 (USP7) and herpesvirus proteins across alpha-, beta-, and gammaherpesviruses. Evidence badges indicate the primary support in the cited studies: S, structural interface mapping; B, direct binding demonstrated with purified components; I, interaction detected in cells (e.g., co-IP/AP-MS and/or colocalization); F, functional phenotype reported in the experimental system. USP7 domain annotations indicate cases where studies mapped binding to specific USP7 regions. Created in BioRender. Hasselmann, T. (2026) https://biorender.com/0xwldon.

**Figure 3:**
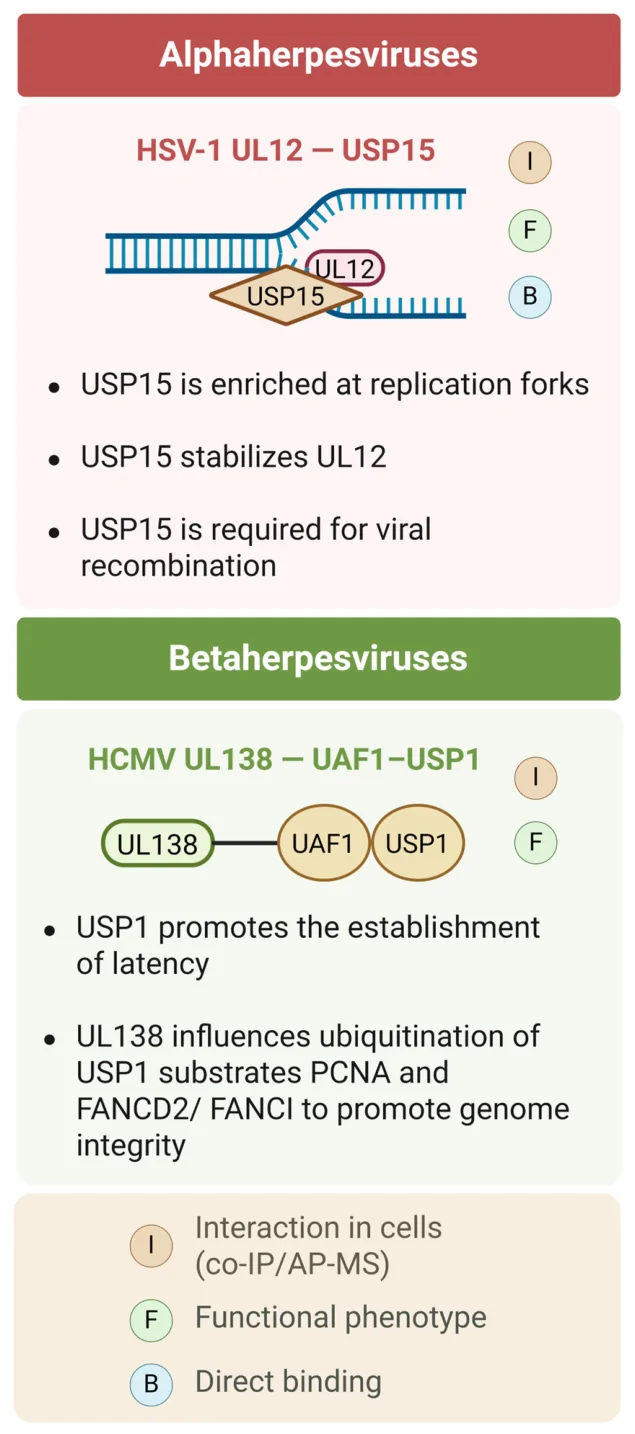
Validated herpesvirus–host USP interfaces highlighted in this review. Schematic summary of experimentally supported interactions between herpesvirus proteins and host ubiquitin-specific proteases (USPs) other than USP7, grouped by herpesvirus subfamily. Evidence badges indicate the primary support in the cited studies: B, direct binding demonstrated with purified components; I, interaction detected in cells (e.g., co-IP/AP-MS and/or colocalization); F, functional phenotype reported in the experimental system. Created in BioRender. Maarouf, M. (2026) https://biorender.com/nxrtpzc.

The viral interactions with USP7 are highlighted in Figure 2. Other USPs are highlighted in Figure 3 and Table 1.

## 4. Herpesvirus-Encoded DUBs

All herpesviruses encode a USP encoded at the N-terminus of the large tegument protein that bears no homology to known DUBs or Ub binding proteins. This was first described for HSV-1 UL36 called UL36^USP^. The enzymatic activity of UL36^USP^ peaks at late times post-infection and appears to require proteolytic cleavage from the rest of the large tegument protein [87]. Other herpesvirus large tegument proteins have very low sequence identity with UL36^USP^, but all contain the conserved residues of the cysteine protease active site. The purification of the EBV and MCMV USPs reveal that they all retain ubiquitin protease activity [88].

There have been several reported activities for HSV-1 UL36^USP^. One major target of UL36^USP^ is the host protein PCNA. UL36^USP^ reduces PCNA ubiquitination, inhibits Pol η foci formation, and increases cellular sensitivity to DNA damage [89]. UL36^USP^ also deubiquitinated PCNA more efficiently than USP1/UAF1 [89]. Another host target of UL36^USP^ is TRAF3. The expression of UL36^USP^ disrupts the TRAF3-dependent recruitment/activation of TBK1 and interferes with IFN-β signaling, and an HSV-1 ΔUL36^USP^ virus induces higher IFN-β production [90]. UL36^USP^ also targets IκBα to reduce NF-κB activation and downstream antiviral gene induction [91]. Similarly in animals, HSV-1 ΔUL36^USP^ virus induced higher levels of IFN-β and IFN-stimulated genes (ISGs) in the brain, and identified STING as the target of UL36^USP^, supporting a role for UL36^USP^ in immune evasion [92]. UL36 was also reported to have an immunomodulatory function independent of its DUB activity, via binding to the IFNAR2 subunit, blocking JAK1–IFNAR2 interaction, and dampening JAK–STAT antiviral signaling, thereby inhibiting IFN-β–induced ISG transcription [93]. Finally, PRV UL36 was shown to be important for initial neural invasion, but not for intraneural transmission [94].

The HCMV homolog of HSV-1 UL36 is UL48. UL48 is also a large tegument protein with an N-terminal USP domain, a conserved active site, and auto-deubiquitinating activity [95,96]. UL48, together with its interacting partner UL45, interacts with and deubiquitinates RIP to inhibit NF-κB signaling [97]. During infection with a UL48 active-site mutant virus, the most affected proteins were UL48 itself and the inner tegument protein pp150 [98]. UL48 also modulates pattern-recognition receptor-mediated type I interferon induction by deubiquitinating TRAF6, TRAF3, IRAK1, IRF7, and STING [99]. The murine cytomegalovirus DUB, M48, is required for the regulation of the viral chemokine, MCK2. The loss of M48 results in elevated levels of MCK2 and unconstrained viral induced inflammation that inhibits replication [100].

The large tegument protein of EBV, BPLF1, also encodes an N-terminal DUB that targets PCNA for deubiquitination [101]. Similar to HSV-1 UL36^USP^, the expression of the BPLF1 DUB is sufficient to prevent PCNA ubiquitination after DNA damage and prevent recruitment of the TLS polymerase η, suggesting a common feature several herpesviruses need to avoid. Several of the N-terminal DUBs also contain PCNA-interacting peptides, or PIP domains, that regulate their interaction with PCNA [101]. BPLF1 also targets and stabilizes the E3 Ubiquitin ligase that monoubiquitinates PCNA, Rad18. A BPLF1 knockout virus had reduced levels of Rad18 upon reactivation [102]. The KSHV large tegument protein, ORF64, also contains N-terminal DUB activity and is required for efficient viral replication and reactivation from latency [103]. KSHV ORF64 counteracts RIG-I/IFN signaling by reversing TRIM25-mediated K63-linked RIG-I ubiquitination, a key step in RIG-I activation [104]. The inhibition of RIG and type I interferon induction seems to be another conserved function of the herpesvirus DUBs.

## 5. Proteomic Maps of USPs Remodeling in Herpesvirus Infection

Leveraging large-scale proteomics in the context of herpesvirus infections could help identify candidate host or viral factors that promote viral growth or act as antivirals. Two complementary classes of datasets have been reported for multiple herpesviruses that we will analyze to try to identify understudied USPs with altered accumulation levels following infection: (i) time-resolved whole-cell proteomics that quantifies USP abundance over a defined time post-infection; and (ii) proteomics identifying host factors physically present on viral DNA. Implementing these two approaches can help distinguish USPs whose total cellular abundance changes during infection (which may reflect destabilization or transcriptional downregulation) from USPs enriched on viral DNA, which is more suggestive of a direct role at viral DNA replication sites.

### 5.1. Time-Resolved Whole-Cell Proteomics

An early study utilized multiplex tandem mass tag (TMT)-based quantitative temporal viromics to profile proteomic changes during HCMV infection over time [105]. The data demonstrate that USP1, USP10, USP22, and USP36 exhibited an early increase (peaking 24 hpi) followed by reduced abundance at later time points (72–96 hpi) relative to mock, whereas USP13 showed an overall decrease from mock across the time course. USP4 was generally reduced post-infection, while USP20, USP33, and USP7 showed increased abundance during infection time points [105].

Follow-up studies used a similar approach with TMT-based temporal proteomic analysis of HSV-1 infection using whole-cell lysates [106]. Of the DUBs observed in this study, USP1 and USP33 were downregulated by 90% and 75%, respectively. They also observed intermediate downregulation of about 40% to 50% for USP7, USP11, USP22, and USP20. USP53 and USP54, which are often annotated as catalytically inactive DUBs, were likewise reduced at late time points [106].

These approaches have also been used to study changes in abundance and posttranslational modifications of host proteins during a time course of HSV-1 infection [107]. USP7 abundance decreased over the time course, while USP5 increased at late time points. USP10, USP24, and USP53 were also reduced over time. They also quantified host phosphosite dynamics, and observed phosphopeptide abundance increased over time for USP15, USP24, USP53, and USP9X. USP34 and USP10 also exhibited increases in phosphorylation at late times post-infection. Conversely, phosphosites on USP8 were downregulated during infection [107].

Another study used TMT-based phosphoproteomic profiling with matched total-proteome analysis in HSV-1-infected cells at 3 hpi. In the total-proteome dataset, USP9X and USP7 showed only modest decreases. In the phosphoproteomic dataset, several USP phosphosites also changed modestly, including decreases in USP8, USP36, and USP10, and increases in USP39 and USP34 [108].

### 5.2. Host Proteins Physically Present on Viral DNA

Another approach to understand virus biology has involved the application of isolation of proteins on nascent DNA (iPOND) coupled with quantitative mass spectrometry. This technique allows for the identification of proteins physically associated with replicating DNA, and preferentially identifies proteins involved in the transcriptional control of viral genes and in the replication of viral DNA. Several labs have utilized iPOND-MS to identify proteins associated with viral DNA during HSV-1 infection [65,109,110]. Most of the observed USPs were common between virus- and host-replicating DNA, but several were either enriched or under-represented on viral DNA [65]. In particular, USP1, USP10, USP3, USP47, USP48, and USP9X were under-represented after infection with HSV-1, while USP15 and USP39 were greatly enriched [65]. Another approach utilized accelerated native iPOND (aniPOND) to demonstrate that USP39 associates with the incoming viral genome as early as 1 h post infection, and remains associated through the start of DNA replication at 6 h post infection [111].

The application of iPOND has also been utilized to study the viral changes associated with loss of viral proteins or in the presence of small-molecule inhibitors. The comparison of HSV-1 versus ΔICP0 virus was used to identify novel viral restriction factors such as SLFN5. The authors also note that USP7 is enriched on viral DNA in the absence of ICP0 [112]. USP34 and USP48 showed a lower abundance in WT relative to mock and ΔICP0 conditions, whereas USP39 was enriched in WT relative to mock. USP15 showed a higher abundance in ΔICP0 relative to WT and USP1 appeared reduced in ΔICP0 versus WT [112]. The comparison of HSV-1 versus ΔUL12 virus also used iPOND to confirm the recruitment of USP15 to viral DNA observed in other studies, and demonstrate that it was recruited specifically by UL12 [68]. Finally, aniPOND was also used to demonstrate that USP39 is still enriched on viral DNA in the presence of PCNA inhibitors [113].

While changes in USP1 and USP7 have been experimentally demonstrated to be important for several herpesviruses, the other identified understudied USPs highlighted by these datasets remain candidates that require further investigation. Future work will be required to determine whether the enriched USPs highlighted by these datasets have direct roles in in the viral life cycle, or if USPs that are lost or reduced in abundance represent novel restriction factors.

## 6. Therapeutic Opportunities

The Ub-proteasome system has been proposed as a drug target, and there are currently three FDA-approved drugs that target the proteasome (bortezomib, carfilzomib, ixazomib). The targeting of individual USPs is still in its early stages, and has only started yielding human clinical trial data in a small set of studies [114].

In our current review of herpesvirus–USP interactions, we highlighted USP1 and USP7 as interacting partners for multiple herpesviruses, and USP1 and USP7 have been proposed to have clear translational potential as drug targets. Since USP1 has been reported to be directly involved in DDR pathways [23,115], targeting it may narrow its antiviral applications due to potential side effects. The USP1 inhibitor TNG348 is a potent, highly selective oral allosteric USP1 inhibitor from Tango Therapeutics. It entered Phase 1/2 testing, but the trial was terminated after significant hepatotoxicity/liver function abnormalities (grade 3/4) were observed in initial cohorts after >8 weeks of dosing [116]. A Roche-sponsored USP1-targeting drug, KSQ-4279/RO7623066, is being evaluated in a Phase 1 clinical study in advanced solid tumors as monotherapy and in combination regimens with carboplatin or olaparib [117,118]. KSQ-4279 is reported to bind to a cryptic pocket in USP1 [118]. Whether the USP1–herpes interaction pocket overlaps with this site is an open question. It remains to be seen whether USP1 inhibition could disrupt a herpesvirus–USP1 axis without triggering unacceptable genotoxic stress.

USP7 is another example of a druggable target given its crucial role in regulating p53 levels [119]. Despite several small molecules showing efficacy in vitro in inhibiting USP7, there are currently no clear active clinical trials testing USP7 inhibitors. For instance, FT671 was described as a selective USP7 inhibitor, and its application exhibits a similar biology to the genetic loss of USP7. For example, FT671 increased p53 levels, destabilized MDM2, induced p21, and exhibited in vivo anti-tumor activity [120]. YCH2823 was reported as a next-generation inhibitor of USP7, surpassing FT671 potency by approximately 5-fold [121]. Assuming no adverse safety profiles of USP7 inhibitors, countering USP7 may be considered as an anti-herpesvirus drug with the potential to target multiple viruses. USP7 inhibition would, in principle, interfere with HSV-1 ICP0-USP7 and HCMV UL35/UL35a-USP7 pathways. However, since USP7 is involved in cell-cycle control via the p53–MDM2 axis and other DDR networks, dosing and timing could be critical to achieving antiviral efficacy with minimal cell-cycle disturbance.

## 7. Conclusions and Open Questions

While targeting host USPs is in the spotlight, another complementary approach would be targeting viral DUBs to ensure higher drug selectivity, while research into herpesvirus DUB inhibitors is relatively scarce [122]. In one reported study, a small-molecule screen identified 10 compounds that inhibited EBV BPLF1 deubiquitinating activity in a pilot assay [123]. Suramin was found to be the top hit, decreasing EBV infectivity in vitro, despite not being selective for the viral DUB. It also inhibited the KSHV DUB, ORF64, in vitro, but did not alter KSHV infectivity or reactivation [123]. This helps establish a framework for targeting viral DUBs, but it also underscores the need to achieve true selectivity while avoiding the cross-inhibition of host USPs. In addition, because many herpesviruses switch between lytic infection and latency, targeting either host USPs or viral DUBs could risk unintended outcomes on genome stability and immune signaling, which is particularly concerning in patients exposed to DNA-damaging agents or in EBV/KSHV-associated malignancies treated with genotoxic chemotherapy. Yet, accumulated knowledge from targeting DUBs in cancer suggests that utilizing similar approaches as antivirals will likely require tight dosing windows with biomarkers for target engagement, so as to reduce inhibitor exposure while maximizing efficacy [114].

## Figures and Tables

**Table 1:** Table summarizing viral proteins interacting with USPs.

Herpesvirus	Viral Protein(s)	Host USP(s)	Interaction	Ref.
HSV-1	ICP0	USP7	USP7 stabilizes ICP0	[60]
	ICP0	USP9X	USP9X is antiviral and negatively regulates ICP0	[82]
	UL12	USP15	USP15 stabilizes UL12 and is required for viral recombination	[68]
PRV	VP16	USP14	USP14 inhibition reduced PRV replication	[83]
HCMV	UL35/UL35a	USP7	UL35/UL35a bind USP7; UL35 relocalizes USP7	[70]
	UL138	USP1	UL138 binds UAF1-USP1 to promote latency and regulate DDR	[71,72]
EBV	EBNA1	USP7	EBNA1 binds USP7 TRAF-like domain; required for EBNA1-mediated PML-NB disruption	[75,76]
KSHV	vIRF1	USP7	vIRF1 identified as a USP7 interactor; dampening p53-mediated antiviral responses	[77]
	vIRF2, vIRF3, vIRF4	USP7	Bind USP7 to regulate innate immune sensing and latency	[79,80,81]
	LANA	USP7	LANA binds USP7; loss of the USP7-binding site increased latent-origin plasmid replication	[84]
	ORF45	USP7	ORF45 binds USP7; linked to stabilization of viral ORF33 and progeny production	[85]
	ORF36	USP9X	Depletion of USP9X inhibited both KSHV reactivation and the production of infectious virions	[86]

## Data Availability

No new data were created or analyzed in this study.

## Abbreviations

The following abbreviations are used in this manuscript:

BRCA1/2	Breast cancer susceptibility gene 1/2
BRCT	BRCA1 C-terminal (domain)
CMV	Cytomegalovirus
Cul4	Cullin 4
DCAF1	DDB1- and CUL4-associated factor 1
DDA1	DET1- and DDB1-associated protein 1
DDB1	Damage-specific DNA binding protein 1
DDR	DNA damage response
DSB	Double-strand break
DUB	Deubiquitinase
EBV	Epstein–Barr virus
EBNA1	Epstein–Barr virus nuclear antigen 1
EGPS	Glu–Gly–Pro–Ser (motif)
E1	Ubiquitin-activating enzyme
E2	Ubiquitin-conjugating enzyme
E3	Ubiquitin ligase
FA	Fanconi anemia
FANCD2/FANCI	Fanconi anemia complementation group D2/I
GMPS	GMP synthetase
Hpi	Hours post-infection
HR	Homologous recombination
HCMV	Human cytomegalovirus
IFN	Interferon
IFNAR2	Interferon-α/β receptor subunit 2
iPOND	Isolation of proteins on nascent DNA
IRAK1	Interleukin-1 receptor–associated kinase 1
IκBα	Inhibitor of κB alpha
ISG	Interferon-stimulated gene
JAMM/MPN^+^	JAB1/MPN/MOV34 metalloenzyme (family)
JAK	Janus kinase
KSHV	Kaposi’s sarcoma-associated herpesvirus
K (e.g., K48, K63)	Lysine (ubiquitin linkage site)
LANA	Latency-associated nuclear antigen
M48	Murine CMV UL48 orthologue
MCK2	Murine CMV chemokine (viral proinflammatory chemokine)
MCM	Minichromosome maintenance (complex)
MCM-BP	Minichromosome maintenance binding protein
MCMV	Murine cytomegalovirus
MDM2	Mouse double minute 2 (E3 ligase regulating p53)
Met1	N-terminal methionine 1 (ubiquitin linkage site)
MINDY	Motif interacting with ubiquitin-containing novel DUB family
NF-κB	Nuclear factor kappa B
NBS1	Nijmegen breakage syndrome protein 1
ORF	Open reading frame
OTU	Ovarian tumor (DUB family)
PCNA	Proliferating cell nuclear antigen
PML	Promyelocytic leukemia (protein)
PML-NB	PML nuclear body
PRV	Pseudorabies virus
RING	Really interesting new gene
S/T	Serine/Threonine
S229, T226 (etc.)	Phosphorylation site numbering (Ser/Thr residue position)
S-phase	DNA synthesis phase of the cell cycle
SSA	Single-strand annealing
STAT1/STAT3	Signal transducer and activator of transcription 1/3
STING	Stimulator of interferon genes
TBK1	TANK-binding kinase 1
TLS	Translesion synthesis
TMT	Tandem mass tag
TRAF3/6	TNF receptor–associated factor 3/6
TRIM25	Tripartite motif-containing protein 25
Ub	Ubiquitin
UAF1	USP1-associated factor 1 (also known as WDR48)
UBL	Ubiquitin-like (domain/protein)
UCH	Ubiquitin C-terminal hydrolase
UL	Unique long (herpesvirus gene region nomenclature)
UPS	Ubiquitin–proteasome system
USP	Ubiquitin-specific protease
vIRF1	Viral interferon regulatory factor 1
VZV	Varicella-zoster virus
WDR48	WD repeat-containing protein 48 (UAF1)
WDR20	WD repeat-containing protein 20
WT	Wild-type
γH2AX	Phosphorylated histone H2AX
ZUFSP/ZUP1	Zinc Finger With UFM1-Specific Peptidase Domain Protein
Zn^2+^	Zinc ion
